# Ethical Issues Associated With Direct-to-Consumer Genetic Testing

**DOI:** 10.7759/cureus.39918

**Published:** 2023-06-03

**Authors:** Kirpal S Panacer

**Affiliations:** 1 Emergency Medicine, Middlemore Hospital, Auckland, NZL

**Keywords:** genetic test kits, genetic ethics, snp-chip genotyping, direct to consumer testing, genetic testing

## Abstract

Direct-to-consumer genetic testing (DTC-GT) is becoming an increasingly profitable private enterprise that provides genetic testing kits directly to consumers. DTC-GT companies advertise themselves as a method for patients to take control of their own health and investigate their risk of diseases and conditions as well as look into their ancestry. The scope of practice of these companies continues to widen offering more services. Consumers may therefore have a relatively poor understanding of the services provided when purchasing these products. The testing methods utilised show some limitations, the consequences of which have the possibility of leading to harm to consumers. The result of the data gathered may instigate the formation of negative stereotypes from the public and reinforce existing ones towards a population that may have already been previously subjugated to unfair treatment. The controversy surrounding how data are utilised further impacts how many may engage in its use. This review aims to provide an overview of the services these companies purport to provide as well as highlight important ethical issues of the service such as quality of information, privacy concerns, negative psychosocial impact and the effect on clinical practice.

## Introduction and background

There are many private companies offering test kits using saliva samples that provide genealogical information screening for specific disease traits. These kits can be easily purchased online, and it has been common to see spikes in purchases during sale times throughout the year. Since 2019, there have been more than 26 million people that have been tested by the four largest direct-to-consumer genetic testing (DTC-GT) companies [1].

Different types of direct-to-consumer genetic tests provide screening for different types of information, these include carrier screening, potential health risk, potential pharmacological intolerance/allergy risk, predisposition to cancer, ancestry and uninterpreted raw genetic data [2].

## Review

Data quality

Genetic test kits advertised by DTC-GT companies are marketed and sold as being able to provide consumers with information regarding potential risks for disease. The method most commonly utilised for analysing DNA is single nucleotide polymorphism (SNP)-chip genotyping which demonstrates high accuracy when analysing the presence of common SNPs of disease. Results of genetic testing are compared against genome-wide association studies (GWAS) data which provides information on whether an SNP is statistically associated with traits and diseases. Although disease associations may exist, phenotypic expression of disease may not necessarily occur. An example highlighting this includes the G-174C polymorphism on the interleukin gene. This polymorphism is associated with many diseases including cardiovascular diseases; however, a meta-analysis consisting of a large number of patients demonstrated that its presence had no clinically significant effect on the risk of cardiovascular diseases [3]. The sensitivity and specificity of SNP-chip genotyping significantly declines as allelic frequency decreases making assessment of rare variants increasingly inaccurate. This can result in a significant proportion of false positives and clinically irrelevant results. An example of poor accuracy of SNP-chip genotyping seen with very rare genetic variants is BRCA1 and BRCA2 is shown in a study demonstrating a positive predictive value of less than 5% [4].

The option for customers to download uninterpreted genetic data is offered by some DTC-GT companies following receiving their results. This data can be interpreted by third-party services that use genomic browsers and other sources to assess it and provide carrier status for conditions and additional risk scores for diseases and conditions [5]. These genomic browsers from which the data is gathered may offer outdated results or results that require additional unavailable information to be interpreted correctly. The result of this incorrect third-party interpretation has led to an inappropriate increase in clinic appointments from consumers who are unaware of the lack of accuracy of the results they have received [6].

The DTC-GT testing highlights potential disease-predisposing variants but may neglect to identify disease-protective factors that may be present. If selective genes are identified to identify potential causal variants whilst neglecting to identify protective factors, skewed data may be collected which has the potential to be exploited. This can be seen with a study aimed at identifying variants associated with obesity in Samoan populations. rs12513649 was a common variant identified amongst Samoan populations highly associated with raised BMI, yet many possessed a further variant present on the CREBRF gene that provided a protective influence against diabetes [7]. Further issues may arise with testing if an inadequate medical context is provided prior to testing as risk estimation for traits. Common complex diseases show an increased likelihood of expression when associated with a strong family history [8]. Significant variation in results also occurred across different companies leading to further questions as to the accuracy of the result [1].

Implications of inaccurate results

Although it may be seen as a positive that many of the consumers taking an interest in managing their own health with DTC-GT, inaccurate or poorly interpreted results may lead to potentially harmful consequences. It is important for healthcare providers to be wary of this as it can consequently lead to unnecessary testing and procedures being performed [2,6].

Concerns have also been highlighted regarding the genetic markers used to identify risks of specific conditions. This can be seen with the genetic markers used by 23andMe for BRCA which only identifies variants that are common in Ashkenazi Jews. The statistics show that the variants used to identify the condition were present in approximately 2% of Ashkenazi Jewish females and 0.01% of the general population. The results would miss the BRCA variant in approximately 80% of the population [9]. Customers receiving negative BRCA results outside the Ashkenazi Jewish population may receive false reassurance regarding the risk of breast cancer and neglect to undertake testing via official channels. Third-party interpretation of raw data leads to a significant false positive rate for disease-causing BRCA variations. This was due to the use of gene variants as a marker for disease when their presence was likely benign [10].

The psychological impact of results

The possibility of anxiety and depression following experiencing negative test results with little follow-up support is significant. In a study completed, 38% reported they did not consider the possibility of receiving information regarding ill health [11]. Results indicating increased risk for depression left those consumers with feelings of decreased ability to regulate their mood [12]. Without adequate support, these patients may struggle to cope leading to worsening mental health symptoms.

Results of DTC-GT are usually received through email and without the support of medical professionals to help interpret the results. This can lead to anxiety and without proper expertise to interpret the results, this may be unresolved for some time. Lack of medical knowledge and access to counselling poses a significant issue as anxiety levels were seen to be higher in those who had a poorer understanding of results [13]. This anxiety may be taken forth to the consumer's regular healthcare provider leading to an increased burden on the healthcare system. With inaccurate results, this may also lead to inappropriate surveillance of health [2]. As this is the case with consumers lacking an understanding of the implications of testing, the consent given for the testing may be inappropriate.

Confidentiality and privacy

Many patients decline participation in genomic research due to fears of disclosure of results. Concerns also arise regarding information sharing with pharmaceutical companies and data loss via cybersecurity attacks. A recent study has indicated that 67% of DTC-GT companies provided an insufficient amount of information on how consumer data would be utilised [14]. Although consumers have the right to refuse their data use for research purposes, many customers consent to its use. DTC-GT companies have reported that greater than eight million customers have consented to have their de-identified data used [15]. The consent forms requesting the use of data for research purposes are non-specific in describing who will be using their data and for what purpose. For the DTC-GT company 23andMe, their consent for research document highlights buzzwords in bold that the purpose of their research is ‘to make new discoveries’ and that others ‘may benefit in the future’. On further analysis, the document states that the data may be used by pharmaceutical companies and they do receive sponsorship for data they provide yet are vague in describing who provides this sponsorship and how much income they receive from it. If patients were fully aware of the possible users and uses of their data, they may be less likely to consent [16].

The data protection and privacy legalities have been difficult to find and interpret for consumers when accessing websites providing DTC-GT. A specific area of concern from consumers is not knowing what happens to data in the event of company insolvency. Currently, DTC-GT companies in the United States are currently under no obligation to notify consumers when actioning policy changes of their terms and conditions. This may be concerning to consumers as personal data is being held [17]. Given the information provided to these companies, a resultant lack of trust may ensue due to this. The lack of transparency of these companies on how personal data is being used is ethically questionable.

The results of genomic tests undertaken in Aotearoa New Zealand can legally be requested by insurance companies thus impacting the cost of premiums. Genetic counsellors have a professional responsibility to make patients aware that any testing undertaken in clinics may be required to be disclosed to insurance companies. The limited ability of genetic data to provide information on whether genetic data will result in phenotypic changes may be exploited by insurance providers. The use of genomic data of traits similar amongst specific ethnicities may lead to genomic discrimination amongst populations [18].

Other countries such as the United States, United Kingdom, and Australia have implemented measures to protect patients from insurance provider discrimination based on genetic results. The Genetic Information Non-discrimination Act 2008 developed in the United States provides protection for its citizens. Data protection laws in the United Kingdom protect patients from mandatory disclosure of genetic test results to insurance companies. In 2019, Australia introduced the Moratorium on Genetic Tests in Life Insurance preventing the use of predictive genetic testing for insurance purposes [19]. The lack of genomic data protection for New Zealand residents may lead to a lack of participation with a subsequent lack of genetic research in specific ethnic groups. This is demonstrated by the lack of medical literature representing the Māori population as well as the decline of participation and representation of ethnic minorities in GWAS [20]. The negative impact of this lack of genetic research could be catastrophic as the potential development of medication targeting regions of DNA shared by specific ethnic groups ceases to be a possibility as an end result. There is hope this may change in the near future as a collaborative alliance named Against Genetic Discrimination Aotearoa (AGendDA) within New Zealand aims to address these disclosure issues [18]. The alliance has been supported by the Breast Cancer Aotearoa Coalition (BCAC) in its pursuit of protection against genomic discrimination [21]. The ethical principles whakapapa, tika, manaakitanga and mana suggested by the Te Mata Ira research team advocate for protection of information as a one of its key issues. Implementation of these principles may increase engagement of Māori communities with genetic research [22].

Informed consent

Poor consumer education prior to testing is a concerning issue. A lack of transparency relating to the accuracy of testing, which demographics are covered, penetrance of diseases, privacy of results and storage of data is apparent. A significant proportion of patients reported not considering receiving a result implicating ill health, thus further demonstrating the lack of understanding prior to testing [1]. Test kits have been used by parents on children who may not have directly consented leading to inappropriate investigations [6]. Validity of informed consent is based on passing the following criteria: has to have been offered the appropriate information to understand risks and benefits, has the capacity to process the information given and the consent has to be given voluntarily. Given the lack of transparency of the companies providing the testing regarding the inaccuracy of testing and implication of the results it can be argued that the consent is not informed.

Health improvement following results

A common reason for undertaking genetic testing was the consumers desire to improve their overall health if they were at higher than average risk of illness than the general public [1]. Greater than 50% of those partaking in one study reported that results implying increased risk of disease would at the least stimulate family discussions [23]. Some consumers reported that they undertook this DTC-GT as it provided them with important information that could be passed on to their health provider to assist in monitoring their health. Some consumers undertook testing to gain information regarding hereditary conditions so they could inform their children [24].

Regardless of the above claims from consumers, no statistically significant change in fat intake or exercise in consumers at short term (three months) or long term (one year) follow ups was seen. Alongside this, consumers demonstrated no significant difference in their undertaking of health screening to monitor their health. This was also seen with those receiving genetic testing result showing an increased risk of cancer not being statistically significantly more likely to engage in cancer screening than the general population [13].

In terms of managing risk for other conditions, such as type 2 diabetes, it has been suggested that weight management of individuals in the population would be enough to negate the risks of expression of the disease making the need for DTC-GT negligible [25]. As most of the genomic data used to inform these tests have been collated from populations of European descent, the applicability of results to those of Māori descent may be limited [26].

Increased burden on healthcare

DTC-GT companies do not offer pre- or post-test genetic counselling leading to poorly informed patients seeking support from elsewhere. This has led to the development of online forums and support groups for consumers to share their results in the hope of receiving support with their results with regards to health implications and support for those who have newly found family members via the ancestry genetic testing. Support in these forums is provided by fellow consumers lacking in medical knowledge which may further lead to confusion and distress [27]. Given this, consumers may decide to discuss their results with their healthcare provider. It has been seen that of those who were comfortable in discussing their results with their doctor, a significant proportion expected advice regarding the management of the given results, and of those, some were doubtful of their doctors’ ability to provide sound advice with the given information [28]. The increased workload would impact doctors working in general practice who may have limited ability to interpret the results and counsel patients on their concerns with the results. Lack of information provided on initial consultation may lead to further consultations [29]. This increased burden with regards to genetic counselling may lead to an increase in referrals to genetic counselling services. Genetic counselling appointments last between 30 and 60 mins and therefore any increase in demand on the service has a significant impact [30]. Significant time and expense may be attributed to consultations regarding DTC-GT based on poor quality tests that have little value in health management leading to a strain on healthcare services.

## Conclusions

DTC-GT has been presented as a method for patients to manage their future health by offering predictions of potential diseases yet comes with major limitations. The potential for deterioration of the mental health of consumers is significant and the absence of any notable lifestyle changes following receiving results points towards the limited real-life applicability of tests. Transparency with regard to data accuracy, validity, and use needs to be addressed across many companies. Clarity and stringency of laws surrounding genetic data need to be provided by many countries. New Zealand laws at present, fail to instate reasonable protection against genomic discrimination resulting in justified fears surrounding disclosure of genetic data. With advocacy groups such as AGenDA and BCAC fighting for change, some of these limitations may be addressed in the near future.

## References

[1] Majumder MA, Guerrini CJ, McGuire AL (2021). Direct-to-consumer genetic testing: value and risk. Annu Rev Med.

[2] Horton R, Crawford G, Freeman L, Fenwick A, Wright CF, Lucassen A (2019). Direct-to-consumer genetic testing. BMJ.

[3] Wade CH, Wilfond BS (2006). Ethical and clinical practice considerations for genetic counselors related to direct-to-consumer marketing of genetic tests. Am J Med Genet C Semin Med Genet.

[4] Weedon MN, Jackson L, Harrison JW (2021). Use of SNP chips to detect rare pathogenic variants: retrospective, population based diagnostic evaluation. BMJ.

[5] Nelson SC, Fullerton SM (2018). “Bridge to the literature”? Third-party genetic interpretation tools and the views of tool developers. J Genet Couns.

[6] Moscarello T, Murray B, Reuter CM, Demo E (2019). Direct-to-consumer raw genetic data and third-party interpretation services: more burden than bargain?. Genet Med.

[7] Minster RL, Hawley NL, Su CT (2016). A thrifty variant in CREBRF strongly influences body mass index in Samoans. Nat Genet.

[8] Horton R, Crawford G, Freeman L, Fenwick A, Lucassen A (2019). Direct-to-consumer genetic testing with third party interpretation: beware of spurious results. Emerg Top Life Sci.

[9] Rebbeck TR, Friebel TM, Friedman E (2018). Mutational spectrum in a worldwide study of 29,700 families with BRCA1 or BRCA2 mutations. Hum Mutat.

[10] Tandy-Connor S, Guiltinan J, Krempely K (2018). False-positive results released by direct-to-consumer genetic tests highlight the importance of clinical confirmation testing for appropriate patient care. Genet Med.

[11] Roberts JS, Gornick MC, Carere DA, Uhlmann WR, Ruffin MT, Green RC (2017). Direct-to-consumer genetic testing: user motivations, decision making, and perceived utility of results. Public Health Genomics.

[12] Lebowitz MS, Ahn WK (2018). Blue genes? Understanding and mitigating negative consequences of personalized information about genetic risk for depression. J Genet Couns.

[13] Bloss CS, Wineinger NE, Darst BF, Schork NJ, Topol EJ (2013). Impact of direct-to-consumer genomic testing at long term follow-up. J Med Genet.

[14] Christofides E, O’Doherty K (2016). Company disclosure and consumer perceptions of the privacy implications of direct-to-consumer genetic testing. New Genet Soc.

[15] andMe Research Innovation (2023). 23andMe Research Innovation Collaborations Program. Collaborations Program.

[16] (2022). 23andMe DNA Genetic Testing & Analysis. Analysis.

[17] Hazel JW, Slobogin C (2018). Who knows what, and when?: a survey of the privacy policies proffered by U.S. direct-to-consumer genetic testing companies. Cornell J Law Public Policy.

[18] Shelling AN, Bicknell LS, Bohlander SS (2022). Genomic discrimination in New Zealand health and life insurance. Agenda: Against genomic discrimination in Aotearoa. N Z Med J.

[19] Tiller J, Lacaze P, Otlowski M (2022). The Australian moratorium on genetics and life insurance: evaluating policy compared to Parliamentary recommendations regarding genetic discrimination. Public Health Res Pract.

[20] Popejoy AB, Fullerton SM (2016). Genomics is failing on diversity. Nature.

[21] (2022). Genetic discrimination a threat to NZ patients. NZ patients.

[22] Hudson M, Beaton A, Milne M. Te Mata Ira. (2017). Guidelines for Genomic Research with Maori, University of Waikato Te Mata Hautū Taketake - Māori & Indigenous Governance Centre.

[23] Cherkas LF, Harris JM, Levinson E, Spector TD, Prainsack B (2010). A survey of UK public interest in internet-based personal genome testing. PLoS One.

[24] Su Y, Howard HC, Borry P (2011). Users' motivations to purchase direct-to-consumer genome-wide testing: an exploratory study of personal stories. J Community Genet.

[25] Regalado A (2019). 23andMe thinks polygenic risk scores are ready for the masses, but experts aren't so sure. https://www.technologyreview.com/2019/03/08/136730/23andme-thinks-polygenic-risk-scores-are-ready-for-the-masses-but-experts-arent-so-sure/.

[26] Elhaik E, Tatarinova T, Chebotarev D (2014). Geographic population structure analysis of worldwide human populations infers their biogeographical origins. Nat Commun.

[27] Basch CH, Hillyer GC, Samuel L, Datuowei E, Cohn B (2023). Direct-to-consumer genetic testing in the news: a descriptive analysis. J Community Genet.

[28] Goldsmith L, Jackson L, O'Connor A, Skirton H (2012). Direct-to-consumer genomic testing: systematic review of the literature on user perspectives. Eur J Hum Genet.

[29] Cohidon C, Cardinaux R, Cornuz J (2021). May direct-to-consumer genetic testing have an impact on general practitioners' daily practice? a cross-sectional study of patients' intentions towards this approach. BMC Fam Pract.

[30] (2018). What is genetic counselling?. https://www.macmillan.org.uk/cancer-information-and-support/worried-about-cancer/causes-and-risk-factors/what-is-genetic-counselling..

